# Integrated analysis identifies CCNA2 as a candidate diagnostic and prognostic biomarker in oral tongue squamous cell carcinoma

**DOI:** 10.1590/1678-7765-2025-0618

**Published:** 2026-03-02

**Authors:** Ceren Gumedag, Sevcan Atay

**Affiliations:** 1 Ege University Faculty of Medicine Department of Medical Biochemistry Izmir Turkey Ege University Faculty of Medicine, Department of Medical Biochemistry, Izmir, Turkey.; 2 Izmir University of Economics Faculty of Medicine Izmir Turkey Izmir University of Economics, Faculty of Medicine, Izmir, Turkey.

**Keywords:** Squamous cell carcinoma of head and neck, CCNA2 protein, human, Biomarkers, Prognosis, Gene expression

## Abstract

**Background:**

Oral tongue squamous cell carcinoma (OTSCC) is an aggressive malignancy with poor prognosis, necessitating reliable biomarkers.

**Methodology:**

Genes with significantly higher expression in OTSCC tumor tissues compared to normal tongue tissues were identified via integrated transcriptomic analysis of seven GEO datasets. To assess their diagnostic and prognostic potential, these genes were further characterized using multi-omic and clinical data from the TCGA-OTSCC and CPTAC-OTSCC cohorts.

**Results:**

A total of 1,117 genes were found to be upregulated in OTSCC tissues, among which only CCNA2 (Cyclin A2) was significantly associated with both reduced overall survival (OS) and disease-free survival (DFS) in the TCGA-OTSCC cohort (n=128), based on Cox proportional hazards regression and Kaplan–Meier analyses. CCNA2 showed moderate prognostic performance (AUC=0.63 for OS; AUC=0.65 for DFS) and was significantly upregulated in higher-grade tumors (p=0.01) and in deceased patients (p=0.03). No somatic mutations or promoter methylation alterations were observed in CCNA2 based on TCGA data. In CPTAC-OTSCC samples (n=18), CCNA2 protein expression was significantly higher in tumor tissues than in non-tumoral tissues (p <0.0001), with a positive correlation between mRNA and protein levels (r=0.56, p=0.01). Both mRNA and protein forms showed strong diagnostic performance (AUC=0.92 and AUC=0.82, respectively), consistent with observations across multiple tumor types. While CCNA2 protein levels showed prognostic relevance for OS (AUC=0.69, p=0.01), the mRNA-based prediction did not reach statistical significance (AUC=0.63, p=0.36). Functional enrichment analysis of CCNA2 co-expressed genes predicted involvement in cell cycle, mismatch repair, and DNA replication pathways. Additionally, protein–protein interaction analysis positioned CCNA2 as a central hub, suggesting its potential role in OTSCC pathogenesis.

**Conclusions:**

These findings indicate that CCNA2 is a promising diagnostic and prognostic biomarker candidate in OTSCC. Given the small size of the CPTAC validation cohort, further studies in larger, independent OTSCC cohorts are warranted to confirm its clinical utility.

## Graphical abstract

**Figure:**
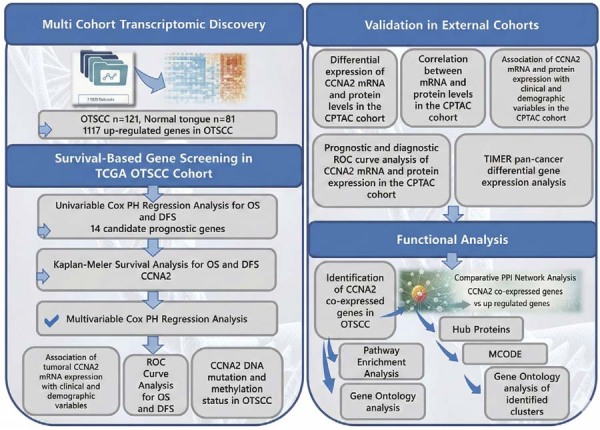

## Introduction

Oral tongue squamous cell carcinoma (OTSCC) represents a distinct anatomical and biological entity within the broader category of oral squamous cell carcinoma (OSCC), which falls under the umbrella of head and neck squamous cell carcinoma (HNSCC). Accounting for nearly 40% of all OSCC cases worldwide, OTSCC constitutes the most common form of oral cavity cancer.^1^ Characterized by an aggressive clinical course, high cellular plasticity, and poor prognosis, OTSCC shows a five-year overall survival rate of about 38%.^2^

Early-stage OTSCC is typically asymptomatic, in which symptoms are often mistaken for minor traumatic lesions, leading to delayed diagnosis and presentation at advanced or metastatic stages, limiting therapeutic options and contributing to poor clinical outcomes.^3^ Tumor volume, lymphovascular invasion, and perineural invasion are recognized as poor prognostic indicators.^4^ Nonetheless, a critical need for reliable molecular prognostic biomarkers to enhance clinical decision-making and optimize treatment strategies remains.

Recent advances in high-throughput sequencing and transcriptomic profiling have greatly expanded our understanding of tumor biology, enabling the discovery of novel molecular biomarkers and therapeutic targets. However, despite extensive molecular research in OSCC and HNSCC, the transcriptomic landscape of OTSCC remains comparatively underexplored. Several transcriptomic studies have proposed candidate prognostic biomarkers for OTSCC, yet most lack independent validation in OTSCC cohorts^5-12^ or clinical translation.^13^ To date, no molecular biomarker has been established for routine clinical use in OTSCC.

In this study, we sought to identify genes that are significantly upregulated in OTSCC compared with normal tongue tissues and to evaluate their potential to serve as both diagnostic and prognostic biomarkers. To the best of our knowledge, this is the first investigation to integrate proteomic and transcriptomic data for biomarker discovery in OTSCC. Furthermore, we mapped the biological pathways and molecular networks associated with these candidate markers to predict their potential roles in OTSCC pathogenesis and to highlight novel therapeutic opportunities.

## Methodology

### Datasets included in the study

Datasets in the NCBI Gene Expression Omnibus (GEO) database were screened using the keywords “oral cancer,” “head and neck cancer,” and “tongue cancer.” The primary inclusion criteria were: (i) tissue samples, (ii) Homo sapiens, and (iii) expression profiling by array. Among the datasets meeting these criteria, only those containing transcriptomic data from at least three OTSCC tumor tissues and three non-tumoral tongue tissues were included.

The prognostic potential of genes upregulated in oral tongue squamous cell carcinoma (OTSCC) was assessed using mRNA expression (RNA-Seq V2 RSEM) and clinical data from the Head and Neck Squamous Cell Carcinoma cohort (TCGA, Firehose Legacy), accessed via cBioPortal (https://www.cbioportal.org/). Only patients with the primary tumor site annotated as “oral tongue” were included, resulting in 133 cases. After excluding three patients with missing mRNA expression data and two patients lacking survival information, the final cohort comprised 128 patients for overall survival (OS) analyses, of whom 101 also had disease-free survival (DFS) data available. This cohort will hereafter be referred to as the TCGA-OTSCC cohort.

Transcriptomic (RNA seq FPKM) and survival data from the CPTAC Head and Neck Squamous Cell Carcinoma (CPTAC-GDC, 2025, n=170) cohort were accessed via the cBioPortal database (https://www.cbioportal.org/). Clinical data, including primary tumor site information, were obtained from the original study by Huang, et al.^14^ (2021), and only patients with the primary tumor site annotated as “tongue” were included (n=20). Protein expression data (TMT log₂ intensity) from tumor and normal tissues were downloaded from the LinkedOmics database (https://linkedomics.org/data_download/CPTAC-HNSCC/). Two patients (C3N-03619 and C3N-03226) lacking protein expression data were excluded. Protein expression profiles were then matched with the patients’ clinical, mRNA, and survival data, and analyses were conducted using this integrated dataset. This cohort will hereafter be referred to as the CPTAC-OTSCC cohort (n=18).

### Identification of up-regulated genes in OTSCC

In the selected GEO datasets, differentially over-expressed genes were identified using GEO2R, with significantly upregulated genes in OTSCC tumor tissues compared to non-tumoral tongue tissues defined by a fold change (FC) ≥ 2 and p ≤0.05. Genes that were consistently upregulated in tumor tissues across at least two independent GEO datasets were selected for further investigation.

### Prognostic significance of upregulated genes in TCGA-OTSCC Cohort

Associations between the expression levels of the identified upregulated genes and OS were first evaluated using univariate Cox proportional hazards regression in IBM SPSS Statistics for Windows (v.28.0.1.1), treating gene expression as a continuous variable. Genes showing a statistically significant association with OS (p≤0.05) were subsequently assessed for DFS using the same Cox regression approach. Hazard ratios (HRs) and 95% confidence intervals (CIs) were calculated for each gene.

To further evaluate the prognostic impact of genes significantly associated with OS or DFS, Kaplan–Meier survival analyses were performed using the custom data analysis option in the Kaplan–Meier plotter (https://kmplot.com/analysis/). Patients were stratified into two groups based on median gene expression to illustrate the effect of high versus low expression on survival, and statistical significance was determined using the log-rank test (p≤0.05). Genes not showing a significant correlation with OS or DFS in Kaplan–Meier analysis were excluded from downstream analyses.

### Evaluation of the prognostic biomarker potential of identified candidate genes in OTSCC

Cox proportional hazards regression analysis was conducted to evaluate clinical and demographic parameters associated with overall survival (OS) and disease-free survival (DFS). Multivariate Cox proportional hazards regression analyses were performed to identify independent prognostic factors for OS and DFS. The proportional hazards assumption was tested for each variable using the Schoenfeld residuals. The associations between mRNA and protein expression levels of the candidate prognostic genes and the clinical or demographic parameters of OTSCC patients were analyzed using unpaired Student’s t-tests, one-way ANOVA followed by Dunnett’s multiple comparison tests, or non-parametric tests when appropriate. The correlation between protein and mRNA expression levels was assessed using Spearman’s rank correlation coefficient. The discriminatory ability of the candidate’s prognostic genes’ mRNA and protein expression levels for OS was evaluated using receiver operating characteristic (ROC) curve analysis. Cox PH regression analyses were conducted in XLSTAT Statistical Software 2025.1.1. All statistical graphs were generated using GraphPad Prism (version 8.0.2; GraphPad Software, Boston, MA, USA), unless otherwise specified. A p-value of ≤0.05 was considered statistically significant in all analyses.

### Timer database analyses

In TCGA cancer cohorts, mRNA expression levels of potential prognostic genes in tumor tissues were compared to non-tumor tissues using the TIMER2.0 database [http://timer.cistrome.org/].

### Pathway enrichment and gene ontology analyses

Genes positively correlated with the identified candidate prognostic genes were identified using mRNA data from TCGA-OTSCC patients (n=130) via cBioPortal. Genes with a Spearman correlation coefficient ≥0.6 and a corresponding p-value ≤0.05 were considered significantly positively co-expressed and reflecting strong associations.^15^ Over-representation analysis (ORA) was conducted to identify Kyoto Encyclopedia of Genes and Genomes (KEGG) pathway categories enriched among genes co-expressed with the identified candidate prognostic genes in OTSCC. Moreover, Gene Ontology (GO) analysis was performed to determine the enriched biological process, cellular component, and molecular function categories among these co-expressed genes. All analyses were conducted using the web-based Gene Set Analysis Toolkit (WebGestalt 2019, [https://www.webgestalt.org/]), with a false discovery rate (FDR) threshold of ≤0.05.

### Protein-protein interaction network (PPI) analysis

The interactions among proteins encoded by the identified up-regulated genes in OTSCC and genes co-expressed with the identified candidate prognostic genes in TCGA-OTSCC cohort were analyzed separately using the STRING database (v11.0), (https://string-db.org). Protein-protein interaction data were retrieved with a confidence score cutoff of 0.4 and a maximum additional interaction value of 0. The interaction network was then visualized using Cytoscape (version 3.10.3). Subsequently, densely interconnected protein clusters within the PPI network were identified using MCODE, a Cytoscape application. The Gene Ontology Biological Process categories enriched in these clusters were then determined via STRING Functional Enrichment Analysis (FDR≤0.05). Nodes with a high degree are typically classified as “hub” proteins in PPI networks, signifying their extensive interaction with other proteins. The degree of a node is defined as the number of edges directly connected to it, representing the extent of its connections within the network. Hub proteins within the overall OTSCC PPI network were identified using Network Analyzer, a CytoScape tool, applying a cutoff criterion of a degree ≥120. The results of the two conducted PPI network analyses were compared to predict the potential importance of the protein network associated with the identified candidate prognostic genes and their co-expressed genes within the overall OTSCC PPI network.

### Mutation and methylation analysis

DNA sequencing data (n=133) and HM450 methylation β-values (n=130) were available from the TCGA-OTSCC cohort via the cBioPortal for Cancer Genomics (https://www.cbioportal.org/). The relationship between mRNA expression (RNA-seq V2 RSEM) and promoter methylation was assessed using Spearman’s rank correlation coefficient. Note that synonymous mutations are not currently supported in cBioPortal. For genes represented by multiple probes, the analysis includes only the probe showing the strongest negative correlation between methylation signal and gene expression.

## Results

### Identification of up-regulated genes in OTSCC

Transcriptomic data from 121 human oral tongue squamous cell carcinoma (OTSCC) tissues and 81 non-tumoral tongue tissues were retrieved from seven GEO datasets (GSE78060, GSE9844, GSE138206, GSE31056, GSE13601, GSE19089, and GSE75538) available in the NCBI Gene Expression Omnibus database (Table 1). Differential gene expression analysis was performed using GEO2R. Supplementary Files 1 and 2 show, respectively, the DEG lists for each dataset, including FDR-adjusted p-values and other statistical details, and the corresponding R scripts for these analyses. Supplementary File 3A shows the final set of 1,117 consistently upregulated genes.

**Table 1:** GEO datasets included in the study.

Dataset ID	Platform	Tumor (n)	Non-tumor (n)
GSE78060	Affymetrix Human Genome U133 Plus 2.0 Array	26	4
GSE9844		26	12
GSE138206		5	5
GSE31056		16	16
GSE13601	Affymetrix Human Genome U95 Version 2 Array	31	27
GSE19089	Illumina HumanHT-12 V3.0 expression beadchip	3	3
GSE75538	Illumina HumanHT-12 WG-DASL V4.0 R2 expression beadchip	14	14

A total of 13 genes showed consistently elevated mRNA levels in OTSCC tissues compared to non-tumoral tongue tissues across all seven datasets. These genes were LAMB3 (*Laminin Subunit Beta 3*), LAMC2 (*Laminin Subunit Gamma 2*), SPP1 (*Secreted Phosphoprotein 1*), ITGA6 (*Integrin Subunit Alpha 6*), MMP12 (*Matrix Metallopeptidase 12*), CDH3 (*Cadherin 3*), EXT1 (*Exostosin Glycosyltransferase 1*), COL4A6 (*Collagen Type IV Alpha 6 Chain*), PTHLH (*Parathyroid Hormone-Like Hormone*), DSG2 (*Desmoglein 2*), OASL (*2’-5’-Oligoadenylate Synthetase Like*), ISG15 (*Interferon-Stimulated Gene 15*), and RBP1 (*Retinol Binding Protein 1*).

### Prognostic biomarker potential of upregulated genes in TCGA-OTSCC cohort

Among the 13 genes commonly upregulated across all datasets, only DSG2 was significantly associated with overall survival (OS) in univariate Cox regression analysis in the TCGA-OTSCC cohort (p≤0.05) (Supplementary File 3B), whereas none of the genes, including DSG2, showed a significant association with disease-free survival (DFS).

Among the identified 1,117 up-regulated genes, Cox Proportional Hazards (Cox PH) regression analysis revealed that the expression levels of 57 genes were significantly associated with overall survival (OS) in the TCGA-OTSCC cohort (n=128), (Supplementary File 3B). Among these, 14 genes were also correlated with disease-free survival (DFS) (p≤0.05), (Supplementary File 3C).

**Figure 1:**
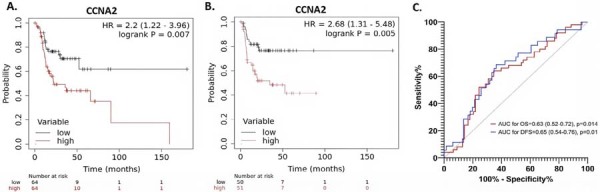
(A) Kaplan-Meier survival curve illustrating overall survival (OS), and (B) disease free survival (DFS) based on CCNA2 mRNA expression levels. The red line represents cases with high expression (expression value > median), while the black line represents cases with low expression (expression value < median). (C) Receiver operating characteristic (ROC) curve showing the area under the curve (AUC) values for overall survival (OS, red) and disease-free survival (DFS, blue) in TCGA-OTSCC patients.

Notably, among the 14 genes that were significantly associated with both overall survival (OS) and disease-free survival (DFS), CCNA2 (Cyclin A2) emerged as the only gene whose high tumoral mRNA expression was strongly associated with poor overall survival (HR=2.2 [1.22–3.96], log-rank p=0.007, Figure 1A) and decreased disease free survival (HR=2.68 [1.31– 5.48], log-rank p=0.005, Figure 1B) as determined by Kaplan–Meier survival curves and Mantel–Cox (log-rank) tests.

ROC curve analysis demonstrated moderate discriminatory power of continuous CCNA2 mRNA levels for predicting both overall survival (AUC for OS = 0.63 [0.52–0.72], p=0.014) and disease-free survival (AUC for DFS = 0.65 [0.54–0.76], p=0.01) in the TCGA-OTSCC cohort (Figure 1C).

### Association of tumoral CCNA2 mRNA expression with clinical and demographic variables in TCGA-OTSCC cohort

In the univariable Cox regression analysis, CCNA2 expression was significantly associated with shorter overall survival (OS) (*p*=0.02; HR=1.0003, 95% CI: 1.000–1.0005) and disease-free survival (DFS) (*p*=0.02; HR=1.000, 95% CI: 1.00–1.001). Hazard ratios correspond to a per unit increase in normalized CCNA2 expression. Among clinicopathological parameters, patients with advanced AJCC T stage (T3–T4) showed markedly poorer OS compared with those with early-stage tumors (T1–T2) (*p*=0.0004; HR=3.018, 95% CI: 1.62–5.61). The association between M stage and survival could not be evaluated, as no patients were classified as M1. Other covariates, including age, sex, AJCC TNM stage, N stage, histological grade, and adjuvant treatments, were not significantly associated with either OS or DFS. (Table 2)

**Table 2:** Clinical and demographic characteristics of the patients from TCGA-OTSCC cohort and the results of the Univariate and Multivariable Cox proportional hazards regression analyses.

Univariable Cox Regression										
Clinicopathological Characteristics	Overall Survival					Disease-Free Survival				
	n	p-value	HR	(95% CI)	PH	n	p-value	HR	(95% CI)	PH
**CCNA2**	128	0.02	1.0003	1.000-1.0005	0.407	128	0.02	1.000	1.00-1.001	0.424
**Age**	128	0.51	1.008	0.98-1.03	0.907	101	0.82	1.003	0.97-1.02	0.413
**Sex**										
Male	83	Reference				68	Reference			
Female	45	0.66	1.138	0.638-2.031	0.441	33	0.47	0.763	0.37-1.59	0.364
**AJCC TNM Stage**										
I-II	34	Reference				30	Reference			
III-IV	82	0.07	2.000	0.92 – 4.32	0.846	62	0.70	1.154	0.54-2.45	0.114
**AJCC T Stage**										
I-II	67	Reference				58	Reference			
III-IV	53	0.0004	3.018	1.62-5.61	0.949	37	0.12	1.711	0.86-3.39	0.077
**AJCC N Stage**										
N0	51	Reference				45	Reference			
I-II	63	0.09	1.725	0.90-3.28	0.894	47	0.55	1.236	0.60-2.50	0.869
**Grade**										
I	17	Reference				13	Reference			
II-III	111	0.26	1.794	0.63-5.05	0.008	88	0.16	2.738	0.65-11.41	0.701
**Pharmaceutical Adjuvant**										
No	26	Reference				22	Reference			
Yes	7	0.18	2.346	0.66-8.33	0,146	6	0.35	1.928	0.48-7.73	0.972
**Radiation Adjuvant**										
No	16	Reference				13	Reference			
Yes	19	0.21	2.313	0.61-8.72	0.994	17	0.16	3.015	0.63-14.22	0.571
**Multivariable Cox Regression Model**										
**Clinicopathological Characteristics**	**Overall Survival**					**Disease-Free Survival**				
	**n**	**p-value**	**HR**	**(95% CI)**	**PH**	**n**	**p-value**	**HR**	**(95% CI)**	**PH**
**CCNA2**	120	0.011	1.000	1.00-1.001	0.541	95	0.03	1.000	1.00-1.001	0.588
**AJCC T Stage**										
III-IV	120	0.0003	3.18	1.69-5.98	0.946	95	0.099	1.787	0.89-3.56	0.082

The proportional hazards (PH) assumption was assessed using Schoenfeld residuals. The assumption held for all covariates (*p*>0.05) except for tumor grade (*p*=0.008), indicating a time-dependent violation of the PH assumption for this variable.

In the multivariable Cox regression model, CCNA2 expression remained independently associated with both OS and DFS after adjustment for AJCC T stage. Elevated CCNA2 levels were linked to increased risks of death (*p*=0.011; HR=1.000, 95% CI: 1.00–1.001) and disease recurrence (*p*=0.03; HR=1.000, 95% CI: 1.00–1.001). Similarly, advanced T stage retained independent prognostic significance for OS (*p*=0.0003; HR=3.00, 95% CI: 1.69–5.98), while its association with DFS did not reach statistical significance (*p*=0.099; HR=1.787, 95% CI: 0.89–3.56). All covariates included in the final model met the PH assumption (all *p*>0.05).

**Figure 2:**
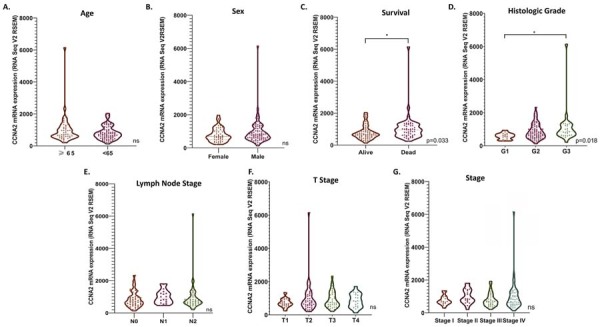
(A-G). Association of tumoral mRNA expression of CCNA2 with clinical and demographic parameters in TCGA-OTSCC patients.

Moreover, the potential association between CCNA2 mRNA expression levels and various clinical parameters, including age, sex, histological grade, lymph node pathological staging, T stage, AJCC tumor stage, and survival status was analyzed in the TCGA-OTSCC cohort (Figure 2 A-G). CCNA2 mRNA expression levels did not significantly differ based on age, sex, lymph node staging, or T stage (p>0.05). However, CCNA2 mRNA expression was significantly elevated in deceased patients (n=50) compared to surviving patients (n=78) (p=0.033), and in poorly differentiated (G3) tumors (n=24) compared to well-differentiated (G1) tumors (n=17) (p=0.018).

### Association of tumoral CCNA2 mRNA and protein expressions with clinical and demographic variables in CPTAC-OTSCC cohort

**Figure 3:**
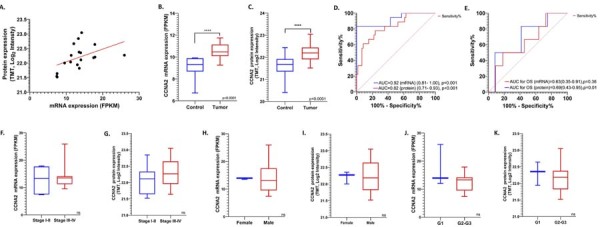
CCNA2 Expression and Its Association with Clinical Parameters in the CPTAC-OTSCC Cohort. (A) Spearman correlation analysis between tumoral CCNA2 mRNA and protein expression levels. Box plots comparing CCNA2 mRNA (B) and protein (C) expression levels in tumor versus non-tumor tissues. ROC curve analysis evaluating the diagnostic (D) and prognostic (E) performances of CCNA2 mRNA and protein levels. Area under the curve (AUC) and statistical significance (p-value) are displayed on the graph. Box plots depicting the relationship between CCNA2 mRNA (F) and protein (G) expression levels and tumor stage. Box plots illustrating the association between sex and CCNA2 mRNA (H) and protein (I) expression levels. Box plots showing the correlation between tumor grade and CCNA2 mRNA (J) and protein (K) expression levels.

In the CPTAC-OTSCC cohort, CCNA2 mRNA levels showed a statistically significant correlation with protein levels (Spearman r=0.56 [95% CI: 0.11-0.82], p=0.01) (Figure 3A). CCNA2 mRNA and protein expression levels were significantly elevated in CPTAC-OTSCC tumor tissues (n= 18) compared to non-tumoral tissues (n=59) (p<0.0001) (Figure 3B and 3C, respectively). ROC curve analysis demonstrated that both CCNA2 mRNA and protein levels possess strong discriminatory power in differentiating OTSCC tissues from normal tissues. The diagnostic performance of CCNA2 mRNA levels was excellent, with an AUC value of 0.92 (95% CI: 0.81–1.00, p<0.001) (Figure 3D). Similarly, CCNA2 protein levels showed a high ability to distinguish tumor tissues, with an AUC value of 0.82 (95% CI: 0.71–0.93, p<0.001) (Figure 3D). Additionally, consistent with the TCGA-OTSCC data, CCNA2 protein levels demonstrated moderate prognostic performance, with an AUC value of 0.69 for overall survival (OS) (95% CI: 0.43–0.95, p=0.01, Figure 3E). CCNA2 mRNA levels showed a trend towards association with OS, but the result did not reach statistical significance (AUC =0.63, 95% CI: 0.35–0.91, p= 0.36, Figure 3E). Furthermore, consistent with TCGA-OTSCC data, no significant association was observed between CCNA2 mRNA or protein expression and tumor stage (Figure 3F and G, respectively) or sex (Figure 3H and I, respectively) in the CPTAC-OTSCC cohort. However, unlike the TCGA-OTSCC data, the previously reported correlation between CCNA2 mRNA expression and tumor grade was not observed in the CPTAC-OTSCC cohort at either the mRNA or protein level (Figure 3J and K).

### Widespread upregulation of CCNA2 in tumor tissues

**Figure 4:**
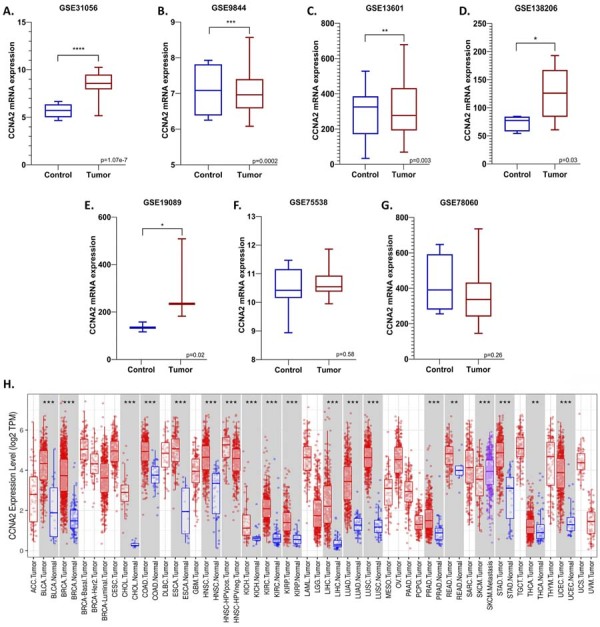
(A-G). CCNA2 mRNA expression was found to be up regulated in five of the seven GEO datasets included in the study. (H) CCNA2 mRNA expressions in various cancer types in the TCGA database were analyzed with TIMER by comparing tumor levels versus non-tumor tissues. (Wilcoxon analysis, **p <0.01, ***p <0.001).

CCNA2 mRNA expression was significantly upregulated in tumor tissues compared to non-tumoral tissues in five out of the seven GEO datasets included in this study (GSE31056, GSE9844, GSE13601, GSE138206, and GSE19089), with a fold change (FC) ≥ 1.5 and a p-value ≤ 0.05 (Figure 4A-G).

To further explore the diagnostic relevance of CCNA2, its mRNA expression was analyzed across multiple human cancer types using the TIMER database. CCNA2 was found to be significantly upregulated in tumor tissues across multiple TCGA cancer cohorts, including BLCA, BRCA, CHOL, COAD, ESCA, HNSC, KICH, KIRC, KIRP, LIHC, LUAD, LUSC, PRAD, READ, STAD, THCA, and UCEC, compared to their respective non-tumoral counterparts (p≤0.05), (Figure 4H).

### Mutation and methylation status of CCNA2 in OTSCC

Analysis of CCNA2 DNA sequencing and HM450 methylation data from the TCGA-OTSCC cohort revealed no somatic mutations in any of the patients. Moreover, tumoral CCNA2 mRNA expression showed no significant correlation with promoter methylation levels (Spearman’s ρ=–0.14, p=0.119; Supplementary Figure 1).

### Functional enrichment analysis of genes Co-expressed with CCNA2 in OTSCC

In the TCGA-OTSCC cohort (n=133), 166 genes were identified as positively correlated with CCNA2 mRNA expression (Spearman correlation ≥0.6, p≤0.05; Supplementary File 3D).

**Figure 5:**
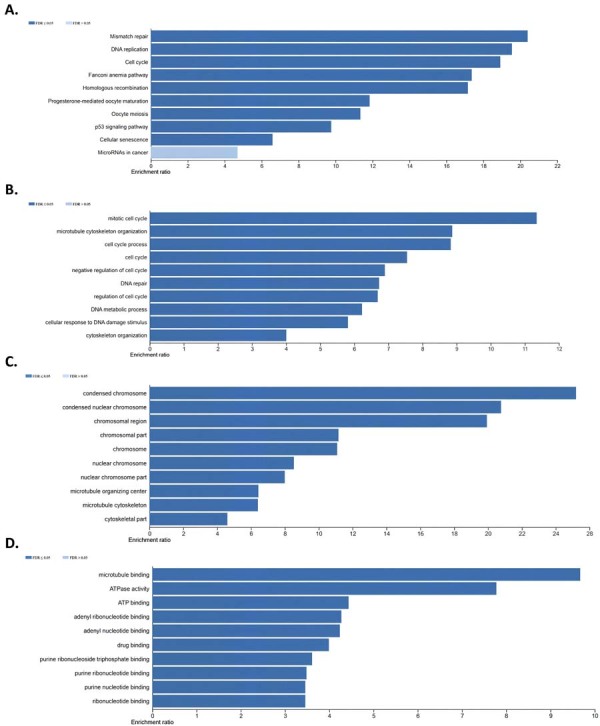
(A) The KEGG pathways enriched by genes co-expressed with CCNA2 in the TCGA-OTSCC cohort, as identified via ORA KEGG pathway analysis and the enriched Gene Ontology (GO) categories, including (B) biological processes, (C) cellular components, and (D) molecular functions are shown. The bar chart tabs are displayed in descending order of enrichment ratio.

The KEGG pathways enriched by genes co-expressed with CCNA2 in the TCGA-OTSCC cohort were predicted using ORA analysis. The results indicated that these genes are involved in key cellular pathways, including “mismatch repair” (FDR=1.3×10^⁻3^), “DNA replication” (FDR=3.12×10⁻⁵), and the “cell cycle” (FDR<2.2×10^⁻16^), (Figure 5A).

Gene Ontology (GO) analysis evaluated the biological processes, cellular components, and molecular functions enriched by genes positively correlated with CCNA2 expression in OTSCC. Gene Ontology (GO) analysis was performed to assess the enrichment of genes positively correlated with CCNA2 mRNA expression in OTSCC across biological processes, cellular components, and molecular functions. The most significant enrichment was observed in the “mitotic nuclear division” biological process (FDR<2.2×10^⁻16^), (Figure 5B), with co-expressed genes predominantly localized in the “kinetochore” component of the cell, (FDR<2.2×10^⁻16^), (Figure 5C) and enriched in the “ATP-dependent activity, acting on DNA” molecular function category (FDR<2.2×10^⁻16^), (Figure 5D). Supplementary File 3E shows the detailed results of all KEGG Pathway and GO enrichment analyses.

### Protein-protein interaction network analysis

**Figure 6:**
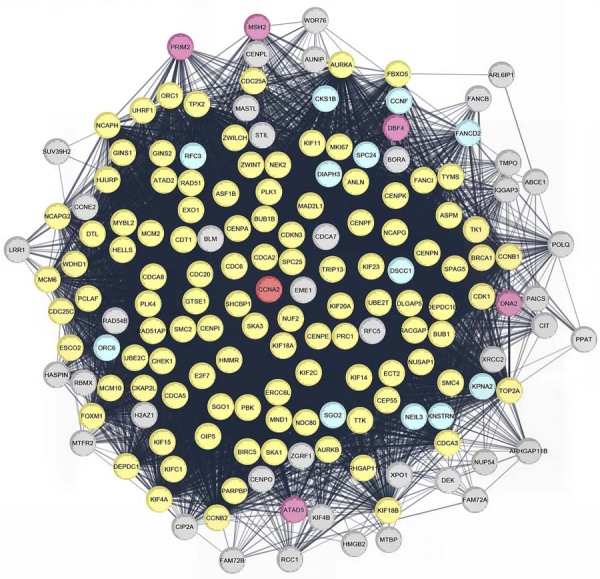
Protein-protein interaction (PPI) network analysis of proteins encoded by genes co-expressed with CCNA2 in OTSCC. The MCODE algorithm identified three distinct protein clusters within the network, which are highlighted in yellow (Cluster 1), pink (Cluster 2), and blue (Cluster 3). CCNA2, positioned as a central hub within the network, is marked in red.

To investigate the molecular context of CCNA2 upregulation in OTSCC, a protein–protein interaction (PPI) network was constructed for the 166 genes positively correlated with CCNA2 in the TCGA-OTSCC cohort (Spearman correlation ≥0.6, p≤0.05; Figure 6). MCODE analysis identified three distinct clusters within this network, colored yellow (Cluster 1), pink (Cluster 2), and blue (Cluster 3). STRING functional enrichment analysis was performed to determine the Gene Ontology Biological Process categories in which the proteins were enriched. Cluster 1 proteins were primarily enriched in “Cell Cycle” (FDR=3.82×10⁻⁸⁶), while Cluster 2 proteins were significantly associated with “Positive Regulation of DNA Metabolic Process” (FDR1.44×10⁻⁵). Cluster 3 proteins showed statistically significant enrichment in the “Mitotic Cell Cycle” process (FDR=1.3 × 10⁻⁴).

A total of 45 hub proteins were identified within the CCNA2 co-expression PPI network (degree ≥ 120), with the top ten including CDK1, BUB1, TOP2A, BUB1B, KIF11, CCNA2, EXO1, TTK, CDCA8, and DLGAP5. Notably, CCNA2 ranked among the top hub proteins with a degree of 141 (Supplementary File 3F).

**Figure 7:**
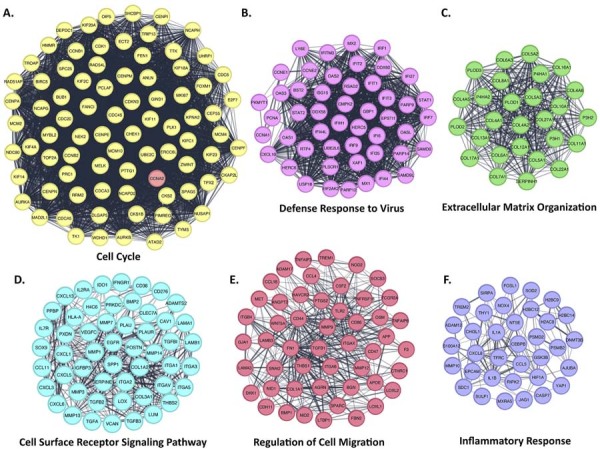
Protein-protein interaction (PPI) network analysis of proteins encoded by up-regulated genes identified in OTSCC. (A-F) The MCODE algorithm identified six distinct protein clusters within the network, highlighted in yellow (Cluster 1), pink (Cluster 2), green (Cluster 3), blue (Cluster 4), red (Cluster 5), and purple (Cluster 6). CCNA2, included in Cluster 1, is marked in red. Enriched Gene Ontology Biological Process categories identified for each cluster are shown below the networks (FDR≤0.05).

Expanding the analysis to the broader OTSCC transcriptome, 1,070 of 1,117 upregulated genes were integrated into a PPI network (Supplementary Figure 2). MCODE identified six distinct clusters within this network (MCODE Score >7). Figure 7 shows that separate PPI interaction networks were constructed for each cluster. Functional enrichment analysis was performed using the STRING Functional Enrichment Analysis tool, revealing the Gene Ontology (GO) Biological Process categories associated with each cluster. Cluster 1, containing CCNA2, enriched in proteins associated with the Cell Cycle pathway (FDR=2.24×10^⁻62^). The other clusters were enriched as follows: Cluster 2: Defense Response to Virus (FDR=1.88×10^⁻41^), Cluster 3: Extracellular Matrix Organization (FDR=4.17×10^⁻25^), Cluster 4: Cell Surface Receptor Signaling Pathway (FDR=6.3×10^⁻16^), Cluster 5: Regulation of Cell Migration (FDR=5.5×10^⁻16^), Cluster 6: Inflammatory Response (FDR=3.23×10^⁻9^).

Within the overall upregulated OTSCC PPI network, 17 hub proteins were identified (Supplementary File 3G), including EGFR, FN1, IL1B, MMP9, CD44, TGFB1, HIF1A, CDK1, CXCL8, STAT1, CCNB1, COL1A1, AURKA, CXCL10, CCNA2, IL1A, and TOP2A. Comparison with the CCNA2 co-expression network revealed CDK1, TOP2A, CCNA2, CCNB1, and AURKA as common hub proteins, all positioned within Cluster 1 in both networks. These findings highlight the potential involvement of these proteins in cell cycle-related processes in OTSCC and suggest they may represent candidate targets for further investigation.

## Discussion

Oral cancer, which ranks among the top ten most common cancers worldwide, primarily refers to malignancies of the oral cavity and oropharyngeal region, with over 90% classified as oral squamous cell carcinoma (OSCC).^16,17^ The Surveillance, Epidemiology, and End Results (SEER) data show that the five-year survival rate for oral cavity and pharyngeal cancers was 69.5% between 2015 and 2021, while age-adjusted incidence and death rates have increased annually by 1.0% and 0.7%, respectively, in the past decade, highlighting persistent challenges in disease prevention and long-term management.^18^ Owing to their distinct etiologies, epidemiological patterns, risk factors, and prognostic outcomes, cancers of the oral cavity and the oropharynx are increasingly recognized as separate disease entities.^19-22^ However, advanced molecular analyses and clinical reports suggest that the heterogeneity of oral cancers extends beyond this anatomical distinction. Primary tumors arising from different anatomic subsites within the oral cavity show distinct transcriptomic,^23^ genetic,^24^ and epigenetic^25^ profiles. These molecular differences may significantly influence metastasis,^26^ treatment response,^27^ and overall prognosis,^28^ underscoring the need for personalized therapeutic approaches and targeted prevention strategies based on the specific molecular characteristics of each anatomical subsite.

The tongue represents the most frequently affected site in Oral Squamous Cell Carcinoma (OSCC), accounting for about 40–50% of all cases.^29,30^ A 2019 analysis of the SEER database, encompassing data from 20,647 patients with oral cavity cancer, demonstrated subsite-specific variability in survival outcomes, with oral tongue squamous cell carcinoma (OTSCC) associated with the poorest prognosis.^31^ Nonetheless, the prognostic significance of anatomical subsite in OSCC remains controversial, and current clinical guidelines do not support subsite-specific treatment approaches.^24,28,32-36^ Emerging evidence suggests that OTSCC is distinct from other OSCC subsites in terms of epidemiology and etiology;^37-46^ however, the molecular mechanisms driving these differences are not yet well understood. Elucidating the molecular pathogenesis of OTSCC may enable the discovery of novel diagnostic and prognostic biomarkers, as well as targeted therapeutic strategies, ultimately contributing to improved patient outcomes.

Despite increasing interest in transcriptomic biomarker discovery, studies that have externally validated OTSCC-specific candidate biomarkers in independent cohorts remain limited. Fang et al. identified and validated a six-gene invasion-related signature associated with disease-free survival in OTSCC, demonstrating moderate predictive accuracy, with five-year DFS AUCs ranging from 0.64 to 0.66 in the testing and validation cohorts.^11^ However, most available transcriptomic investigations in OTSCC have either lacked external validation or relied on broader OSCC or HNSCC datasets as validation cohorts without subsite-specific separation, which limits the biological and clinical interpretability of their findings.^5-10,12^

This study aims to evaluate the prognostic potential of overexpressed genes in OTSCC by systematically screening all upregulated genes, without restricting the analysis to predefined pathways or gene sets. To enhance the reliability of the findings, only transcriptomic datasets derived from OTSCC and non-tumoral tongue tissues were included in all analyses. Furthermore, the genetic, epigenetic, and proteomic contexts of the identified candidate biomarker were investigated to provide a more comprehensive molecular perspective. Finally, the study explores the potential functional roles of the identified candidate gene and its molecular collaborators in OTSCC pathogenesis, offering insights into novel therapeutic targets and contributing to a deeper understanding of OTSCC molecular mechanisms.

Transcriptomic analysis was performed on seven datasets from the Gene Expression Omnibus database that met the strict inclusion criteria, identifying 1,117 genes upregulated in at least two OTSCC cohorts. Among these, 13 genes (LAMB3, LAMC2, SPP1, ITGA6, MMP12, CDH3, EXT1, COL4A6, PTHLH, DSG2, OASL, ISG15, and RBP1) were consistently overexpressed in OTSCC tissues compared to non-tumoral tongue tissues across all seven datasets. ORA KEGG pathway analysis, using the protein-coding genome as a reference, revealed that these genes were enriched in pathways including ECM-receptor interaction, small cell lung cancer, focal adhesion, human papillomavirus infection, and PI3K-Akt signaling (FDR ≤ 0.05). Cox proportional hazards regression analysis in the TCGA-OTSCC cohort indicated that the mRNA expression of these genes was not significantly associated with overall survival or disease-free survival. While these results suggest that high tumoral expression of these genes may not directly influence prognosis, further studies are needed to explore their functional roles in OTSCC pathogenesis and potential impact on other clinical parameters. Given that diagnostic biomarkers for OTSCC are still limited, the consistent upregulation of these 13 genes highlights them as promising candidates for further validation in larger patient cohorts.

Further survival analysis in TCGA OTSCC cohort revealed that 57 of the upregulated genes were significantly associated with overall survival (OS), and 14 of these were also correlated with disease-free survival (DFS). Among them, CCNA2 (Cyclin A2) emerged as the most promising prognostic candidate, showing associations with both OS and DFS in both univariate and multivariate analysis. ROC curve analysis demonstrated moderate predictive performance, and subgroup analysis revealed that CCNA2 expression was higher in deceased patients and in tumors with poor differentiation (G3). Collectively, these results support CCNA2 as a potential prognostic biomarker in OTSCC.

Cyclin A2 is a well-known member of the cyclin family, regulating both the G1/S and G2/M transitions of the cell cycle via its interactions with CDK2 and CDK1, respectively. Several studies have reported CCNA2 overexpression in OSCC and suggested a potential association with patient prognosis, although its diagnostic or prognostic utility has not been systematically assessed.^47-49^ Moreover, to the best of our knowledge, no study to date has examined the clinical significance of CCNA2 expression specifically in oral tongue squamous cell carcinoma (OTSCC).

In this study, CCNA2 was consistently upregulated in five out of seven GEO datasets. Supporting these observations, CPTAC-OTSCC data confirmed that both mRNA and protein expression levels of CCNA2 were significantly higher in tumor tissues compared to non-tumoral tissues. ROC analyses demonstrated excellent diagnostic performance with AUC values above 0.8 for both mRNA and protein levels in distinguishing OTSCC from normal tongue tissues. TCGA pan-cancer analyses indicated that CCNA2 is widely upregulated across multiple cancer types, suggesting that its elevated expression may reflect general oncogenic processes related to proliferation and cell cycle dysregulation. This broad overexpression could limit its specificity in non-invasive diagnostic applications, such as blood or serum-based assays, in which the cellular origin of the biomarker may be unclear. Nevertheless, tissue-based analyses, including this study, demonstrate that CCNA2 expression can effectively distinguish tumor from non-tumoral tongue tissues, supporting its potential utility as a tissue-based diagnostic biomarker in OTSCC.

Furthermore, a moderate but statistically significant correlation was observed between CCNA2 mRNA and protein levels, underscoring the translational relevance of its expression. Importantly, increased tumoral CCNA2 protein levels were significantly associated with poorer overall survival, further supporting its prognostic value at the protein level. Interestingly, although CCNA2 mRNA expression was significantly associated with survival and tumor grade in the TCGA cohort, these associations were not reproduced in the CPTAC cohort. This inconsistency may be attributed to the limited sample size, especially within low-grade tumor groups, as well as key biological factors, including post-transcriptional regulation, protein stability, and tumor microenvironment effects, emphasizing the need for larger, well-powered validation studies to confirm these findings and fully establish the clinical utility of CCNA2.

Mechanistically, CCNA2 expression showed no correlation with DNA methylation status, suggesting that its regulation in OTSCC is likely driven by transcriptional and post-transcriptional mechanisms rather than epigenetic alterations. The absence of CCNA2 mutations further indicates that the wild-type protein is largely maintained in these tumors. Genes positively correlated with CCNA2 expression were enriched in pathways well established in cancer biology, including DNA replication, mismatch repair, and cell cycle regulation. Protein–protein interaction (PPI) network analysis combined with MCODE clustering identified CCNA2 as a central hub within a module enriched for cell cycle-associated genes. The PPI network derived from upregulated genes in OTSCC highlighted Cluster 1 as the module with the highest connectivity, also showing enrichment for cell cycle-related proteins. These findings suggest that networks involving CCNA2 and other cell cycle regulators may contribute to the molecular landscape of OTSCC, although further functional studies are required to clarify their exact roles. Several proteins, including CDK1, TOP2A, CCNB1, CCNA2, and AURKA, were identified as common hubs in both the CCNA2 co-expression network and the upregulated gene network, pointing to their potential relevance as therapeutic targets, which warrants further investigation.

This study shows several inherent limitations that should be considered when interpreting the findings. First, all analyses were performed retrospectively using publicly available datasets, which may differ in cohort characteristics, sample handling, and experimental platforms, potentially introducing technical and biological variability. Second, sample sizes, particularly in the CPTAC-OTSCC cohort, were relatively small, limiting statistical power and generalizability. Third, no patients in the TCGA-OTSCC cohort were classified as M1, preventing assessment of the relationship between distant metastasis and survival outcomes. Moreover, only a small number of patients received adjuvant chemotherapy, limiting the evaluation of treatment effects on survival. Finally, mechanistic and functional interpretations of CCNA2’s role in OTSCC remain hypothetical and require experimental validation. Future studies with larger, prospective, multi-center cohorts are needed to confirm the diagnostic and prognostic potential of CCNA2 and to further clarify its biological significance.

## Conclusions

Our integrated transcriptomic and proteomic analyses consistently identified CCNA2 as an upregulated gene in oral tongue squamous cell carcinoma (OTSCC) across multiple independent datasets. Elevated CCNA2 expression was associated with poorer overall survival and disease-free survival, while protein-level data further supported its diagnostic and prognostic potential. Functional enrichment and protein–protein interaction analyses positioned CCNA2 as a central hub within cell cycle–related networks, implicating it in key processes of OTSCC pathogenesis.

Collectively, these results indicate that CCNA2 is a promising candidate tissue-based biomarker for both diagnosis and prognosis in OTSCC. This study is hypothesis-generating and provides a foundation for future experimental and clinical investigations to validate CCNA2’s utility and further explore its biological and potential therapeutic significance.
